# Phenethyl isothiocyanate activates leptin signaling and decreases food intake

**DOI:** 10.1371/journal.pone.0206748

**Published:** 2018-11-01

**Authors:** Miho Yagi, Yukiko Nakatsuji, Ayumi Maeda, Hiroki Ota, Ryosuke Kamikubo, Noriyuki Miyoshi, Yoshimasa Nakamura, Mitsugu Akagawa

**Affiliations:** 1 Division of Applied Life Science, Graduate School of Life and Environmental Sciences, Osaka Prefecture University, Sakai, Japan; 2 Graduate School of Integrated Pharmaceutical and Nutritional Sciences, University of Shizuoka, Shizuoka, Japan; 3 Graduate School of Environmental and Life Science, Okayama University, Okayama, Japan; Fundacao Oswaldo Cruz, BRAZIL

## Abstract

Obesity, a principal risk factor for the development of diabetes mellitus, heart disease, and hypertension, is a growing and serious health problem all over the world. Leptin is a weight-reducing hormone produced by adipose tissue, which decreases food intake *via* hypothalamic leptin receptors (Ob-Rb) and the Janus kinase 2/signal transducer and activator of transcription 3 (JAK2/STAT3) signaling pathway. Protein tyrosine phosphatase 1B (PTP1B) negatively regulates leptin signaling by dephosphorylating JAK2, and the increased activity of PTP1B is implicated in the pathogenesis of obesity. Hence, inhibition of PTP1B may help prevent and reduce obesity. In this study, we revealed that phenethyl isothiocyanate (PEITC), a naturally occurring isothiocyanate in certain cruciferous vegetables, potently inhibits recombinant PTP1B by binding to the reactive cysteinyl thiol. Moreover, we found that PEITC causes the ligand-independent phosphorylation of Ob-Rb, JAK2, and STAT3 by inhibiting cellular PTP1B in differentiated human SH-SY5Y neuronal cells. PEITC treatment also induced nuclear accumulation of phosphorylated STAT3, resulting in enhanced anorexigenic *POMC* expression and suppressed orexigenic *NPY/AGRP* expression. We demonstrated that oral administration of PEITC to mice significantly reduces food intake, and stimulates hypothalamic leptin signaling. Our results suggest that PEITC might help prevent and improve obesity.

## Introduction

The prevalence of obesity has been increasing explosively over the past decades, resulting today in over 600 million adults worldwide with a body mass index (BMI) of 30 kg/m^2^ or greater [1,2]. Obesity, a principal risk factor for the development of diabetes mellitus, heart disease, and hypertension, is now becoming a serious health problem all over the world [3]. Obesity arises from a chronic positive energy balance that is often attributed to unlimited access to food and an increasingly sedentary lifestyle on the background of a genetic and epigenetic friability [3]. The development of resistance to leptin, a 16-kDa peptide hormone that controls body weight by reducing food intake and increasing energy expenditure, is a common hallmark of obesity [4–6]. Leptin is mainly secreted by adipose cells in proportion to white adipose tissue mass and conveys an adiposity signal to the brain, particularly the hypothalamus, by binding to the leptin receptor (Ob-Rb) [5,7]. Leptin binding to Ob-Rb triggers activation (phosphorylation at Tyr-1007 and Tyr-1008) of Janus kinase 2 (JAK2), which then phosphorylates Tyr-1141 at the extreme C terminus of the cytoplasmic domain of the receptor. The phosphorylated Tyr-1141 binds the signal transducer and activator of transcription 3 (STAT3) from the cytosol, which becomes phosphorylated on a C-terminal tyrosine, either directly by the receptor or by JAK2. Once phosphorylated, STAT3 causes dimerization and translocation to the nucleus, binding to specific promoter sequences in target genes, stimulation of anorexigenic POMC (proopiomelanocortin) expression and suppression of orexigenic NPY/AgRP (neuropeptide Y; agouti related peptide) expression [8]. Meanwhile, protein tyrosine phosphatase 1B (PTP1B) negatively regulates leptin signaling by catalyzing dephosphorylation of tyrosine residues in JAK2 [9–11]. Importantly, mice with whole body or neuron-specific deletion of PTP1B are hypersensitive to leptin and resistant to diet-induced obesity [12,13]. Furthermore, recent studies have indicated that hypothalamic PTP1B is specifically increased during high-fat (HF) diet-induced leptin resistance [14,15]. Based on these facts, PTP1B is a highly plausible candidate for a leptin resistance factor [16] and inhibiting its activity has emerged as a potential therapeutic strategy to treat obesity by restoring leptin sensitivity [17,18].

Isothiocyanates (ITCs) are characterized by the presence of an–N = C = S group, the central carbon of which is thiol-reactive chemicals that can modify critical cysteine residues on a variety of cellular proteins [19–23]. Structurally diverse ITCs are released from corresponding glucosinolates present in many commonly consumed cruciferous vegetables by enzymatic hydrolysis with either a plant-specific myrosinase or intestinal flora in humans [24]. Numerous studies have shown that ITCs can inhibit both carcinogenesis and cancer growth in a variety of animal models [25,26]. Moreover, recent studies emphasize that modification of cellular proteins *via* direct covalent binding of ITCs for an early event in anticarcinogenic activities such as cell cycle arrest or apoptosis induction [19,21,22,27]. Interestingly, more recently, ITSs have been found to inactivate PTP1B [28–30], which has a reactive cysteine residue at the catalytic center [29]. However, the mechanism underlying these effects was not clarified fully.

**Fig 1:**
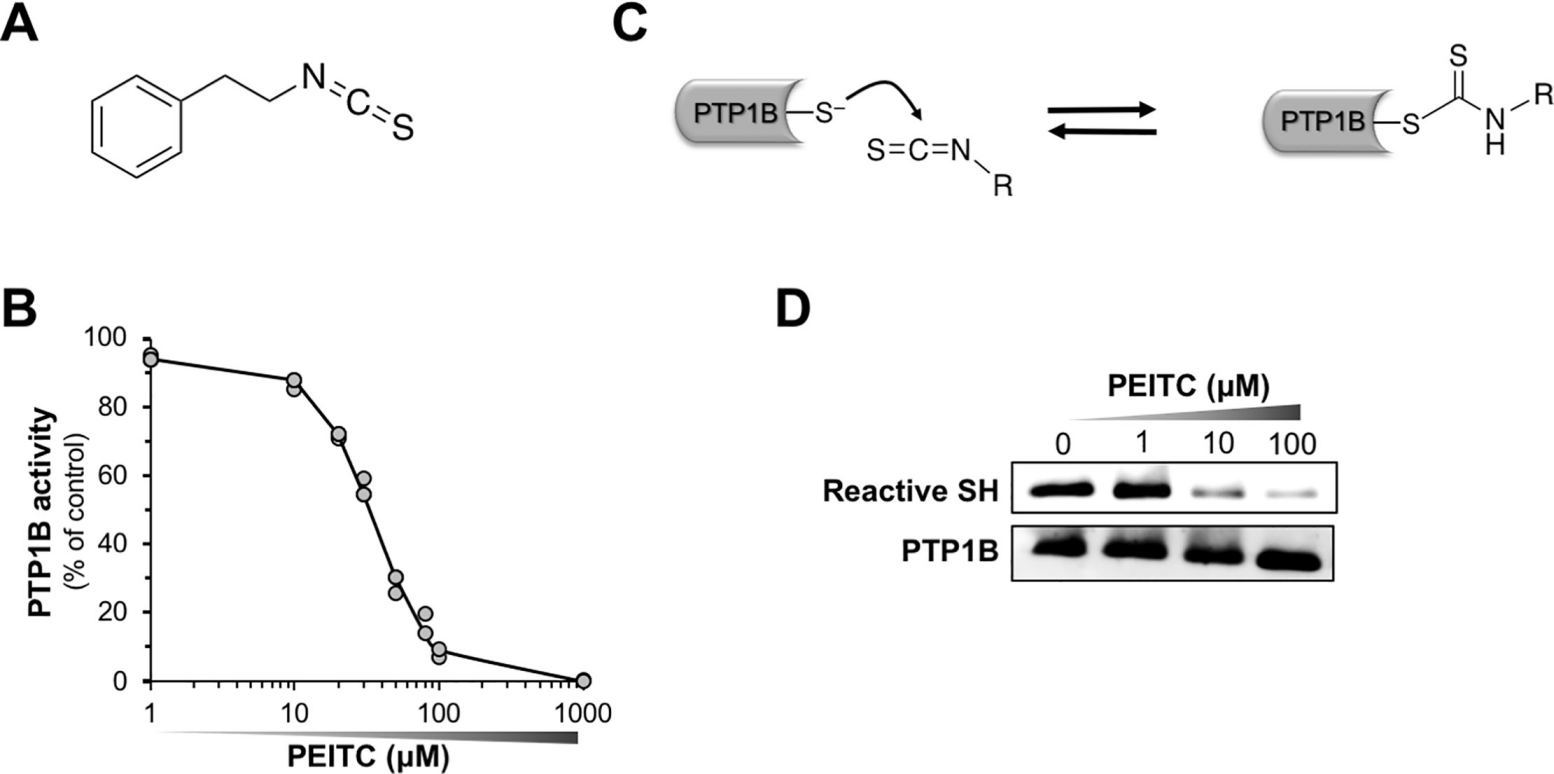
PEITC inhibits PTP1B with concomitant decrease in cysteinyl thiol. (**A**) The structure of PEITC. (**B**) Concentration-dependent inhibition of PTP1B by PEITC. Recombinant PTP1B was incubated with the indicated concentration of PEITC in HEPES buffer (pH 7.5) at 37°C for 30 min, and then PTP1B activity was measured. (**C**) A proposed mechanism for the inhibition of PTP1B through the reaction of ITC with the cysteinyl thiol. (**D**) Concentration-dependent modification of cysteinyl thiol in PTP1B by PEITC. Recombinant PTP1B was incubated with the indicated concentration of PEITC in HEPES buffer (pH 7.5) at 37°C for 30 min. Then, the reactive thiol group and total PTP1B were determined by biotin-labeling assay and immunoblotting, respectively. Data are representative of two independent experiments.

In recent years, there has been increasing interest in the development of ingredients from natural sources for preventing and ameliorating obesity without adverse effects [31–33]. Phenethyl isothiocyanate (PEITC) (**Fig 1A**) is a relatively nontoxic compound that occurs abundantly in water cress and broccoli as gluconasturtiin, and has reached the level of phase 2 clinical trials for lung and oral cancer prevention in the US [34]. After oral administration of [^14^C]PEITC to rats, [^14^C]PEITC and/or its ^14^C metabolites are widely distributed in the tissues including the brain [35]. In the present study, we investigated the inhibitory potency and mechanism of PEITC against recombinant and cellular PTP1B. Furthermore, we found that treatment with PEITC induces a ligand-independent activation of leptin signaling in differentiated human neuronal SH-SY5Y cells. We also demonstrated that oral administration of PEITC to C57BL/6J mice significantly reduces food intake after refeeding. Our results suggest that PEITC might be useful in preventing and reducing obesity.

## Materials and methods

### Materials

Fetal bovine serum (FBS) was obtained from Sigma-Aldrich (St. Louis, MO, USA). Dithiothreitol (DTT), the protease inhibitor cocktail, the phosphatase inhibitor cocktail, Dulbecco's modified Eagle's medium (DMEM), penicillin, streptomycin, Triton X-100, PEITC, allyl isothiocyanate (AITC), fluorescein isothiocyanate (FITC), phenyl isothiocyanate (PITC), and Nonidet P-40 (NP-40) were purchased from Nacalai Tesque (Kyoto, Japan). Sodium orthovanadate (Na_3_VO_4_) and corn oil were purchased from Wako Pure Chemical Industries (Osaka Japan). Benzyl isothiocyanate (BITC) was obtained from Thermo Fisher Scientific (Waltham, MA, USA). All of the other reagents used in the study were of analytical grade and obtained from commercial sources.

### Measurement of PTP1B assay

The recombinant PTP1B activity was assayed using *p*-nitrophenol phosphate (pNPP) as a substrate according to the published procedure [36].

### Detection of reactive cysteine in PTP1B

Reactive cysteine labeling assay was performed according to the published procedure [37]. Iodoacetyl-LC-Biotin and HRP-conjugated streptavidin were obtained from Thermo Fisher Scientific and Beckman Coulter (Fullerton, CA, USA), respectively. *S*-Carbamidomethylated PTP1B was prepared as previously described [38].

### Detection of FITC-modified PTP1B by fluorescence imaging

After the reaction of recombinant PTP1B with FITC, the reaction mixtures were subjected to SDS-PAGE. Then, the fluorescence intensity was detected using a Typhoon FLA-7000 fluorescence imager (Fujifilm).

### Cell culture

SH-SY5Y human neuroblastoma cells (American Type Culture Collection, ATCC, Manassas, VA, USA) were grown in DMEM (Dulbecco’s modified Eagle’s medium) supplemented with 10% heat-inactivated FBS, 100 units/mL streptomycin, and 100 μg/mL penicillin at 37°C in a humidified 5% CO_2_ atmosphere. The cells were differentiated by treatment with 100 nM of all-*trans* retinoic acid (a*t*RA) (Nacalai Tesque) between 1 and 6 days after plating on a collagen-coated culture plate [39]. The differentiation of the cells was confirmed by monitoring neurite outgrowth and by measuring Ob-Rb expression. Differentiated SH-SY5Y cells were washed with serum-free DMEM and starved in serum-free medium. After overnight starvation, the medium was exchanged with fresh serum-free medium, and then the cells were exposed to ITC. Cell viability was measured by WST-8 assay using Cell Count Reagent SF (Nacalai Tesque) following the manufacturer’s instructions.

### SDS-PAGE and immunoblot analysis

SDS-PAGE and immunoblot analysis were performed as previously reported [40]. The anti-phospho primary antibody was diluted with Can Get Signal solution 1 (Toyobo, Osaka, Japan) to enhance the immunoreactions for the phosphorylated proteins. Chemiluminescence was detected using EzWestLumiOne and Luminograph (ATTO, Tokyo, Japan). Quantification of the band intensity was performed using the ImageJ software (National Institutes of Health, Bethesda, MD, USA). Antibodies for the phospho-JAK2 (p-Tyr1007/1008), phospho-STAT3 (p-Tyr705), histone H3, goat anti-mouse IgG-HRP, and goat anti-rabbit IgG-HRP were purchased from Cell Signaling Technology (Beverly, MA, USA). Antibodies for the phospho-Ob-R (p-Tyr1141) GAPDH, and PTP1B were obtained from Santa Cruz Biotechnology (Santa Cruz, CA, USA). Antibodies for the Ob-R, JAK2, and STAT3 were from SIGMA-Aldrich.

### Determination of cellular PTP1B activity

Cellular PTP1B activity was measured by malachite green assay for detection of free phosphate from PTP1B phosphopeptide substrate (Santa Cruz Biotechnology) as previously described [38]. Biomol green reagent was purchased from Enzo Life Sciences, NY, USA.

### Extraction of cytoplasmic and nuclear proteins

The cytoplasmic and nuclear extracts for immunoblotting were prepared as previously described [41]. Protein concentration was measured by Bradford assay (Bio-Rad, Hercules, CA, USA) using BSA as standard according to the manufacturer's protocol.

### RNA isolation and quantitative reverse transcription PCR (qRT-PCR)

The total RNA was isolated from differentiated SH-SY5Y cells using the SuperPrep Cell Lysis Kit for qPCR (Toyobo, Osaka, Japan) according to the manufacturer’s protocol. The total RNA was converted to cDNA using the SuperPrep RT Kit for qPCR (Toyobo). Primers for the human *POMC* (Forward 5'-AGCCCGCCCAAGGACAAG, reverse 5'-TGCCCTCACTCGCCCTTCT), *NPY* (forward 5'-TCCAGCCCAGAGACACTGATT, reverse 5’-AGGGTCTTCAAGCCGAGTTCT), and *AGRP* (forward 5'-CGTCGCTGCGTAAGGCT, reverse 5'-CAGTAGCAGAAGGCATTGAAGAAG) were purchased from Thermo Fisher Scientific. Primers for human *ribosomal protein S18* (*RPS18*) (Primer Set ID: HA067807) were purchased from Takara Bio, Shiga, Japan. qRT-PCR was performed on the Thermal Cycler Dice Real Time System Single (Takara Bio) using the THUNDERBIRD SYBR qPCR Mix (Toyobo). The Ct value was normalized with the housekeeping gene *RPS18* and the relative fold change was computed by the ΔΔCt method.

### Immunocytochemical staining

The immunocytochemical study was performed according to previously described methods [41]. Antibodies for the phospho-STAT3 (p-Tyr705) was purchased from Cell Signaling Technology. The Cy3 conjugated anti-rabbit IgG antibody was obtained from GE Healthcare UK Ltd. (Buckinghamshire, UK). After immunostaining, the cells were mounted with Fluoro-KEEPER Antifade Reagent containing DAPI (Nacalai Tesque), and were analyzed using a fluorescence microscope, BZ-9000 (Keyence, Osaka, Japan).

### Animal experiments

Six-week-old male C57BL/6J mice were purchased from Kiwa Laboratory Animals (Wakayama, Japan). All mice were individually housed at 24 ± 2°C under 12:12-h light-dark cycle with lights on 7:00 AM-7:00 PM, and had *ad libitum* access to water and a standard powdered diet (MF, Oriental Yeast, Tokyo). Animal experiments were carried out according to a protocol approved by the Animal Care Committee of Osaka Prefecture University (permission number: 28–184).

### Effects of oral PEITC administration on food intake and body weight

After 1 week of environmental acclimation, the mice were fasted for 6 h (food removed at 12:00 AM). At 6:00 PM, the mice were divided into 2 groups and given either corn oil (vehicle) or 2.5 mg/mL PEITC-corn oil (25 mg/kg PEITC) *via* oral gavage (10 mL/kg body weight), followed by further 1-h fasting. At 7:00 PM, the mice were allowed *ad libitum* access to a standard powdered diet. After feeding at 0 h (at 7:00 PM), 1 h (at 8:00 PM), 4 h (at 11:00 PM), 14 h (at 9:00 AM), and 24 h (at 7:00 PM), food intake and body weight were measured. In addition, after oral dosing at 2 h, the blood sample was obtained from tail veins, and plasma leptin concentrations were measured using a Murine Leptin Standard ABTS ELISA Development Kit (PeproTech, Rocky Hill, NJ, USA).

### Effects of oral PEITC administration on leptin signaling in the hypothalamus

The mice were orally infused with either corn oil (vehicle) or 2.5 mg/mL PEITC-corn oil (25 mg/kg PEITC) as described above. After further 3-h fasting, the mice were sacrificed by cervical dislocation. Then, the hypothalamus was quickly dissected out, frozen in liquid nitrogen, and kept at –80°C. The frozen hypothalamus was homogenized in radio-immunoprecipitation assay (RIPA) buffer containing 1% Triton X-100, the phosphatase inhibitor cocktail, and protease inhibitor cocktail using a bead-beater type homogenizer. The homogenates were centrifuged at 13,000 × *g* for 15 min at 4°C, and the supernatants were analyzed by immunoblotting as described above.

## Results

### PEITC inhibits recombinant PTP1B with concomitant decrease in reactive cysteinyl thiol

**Fig 2:**
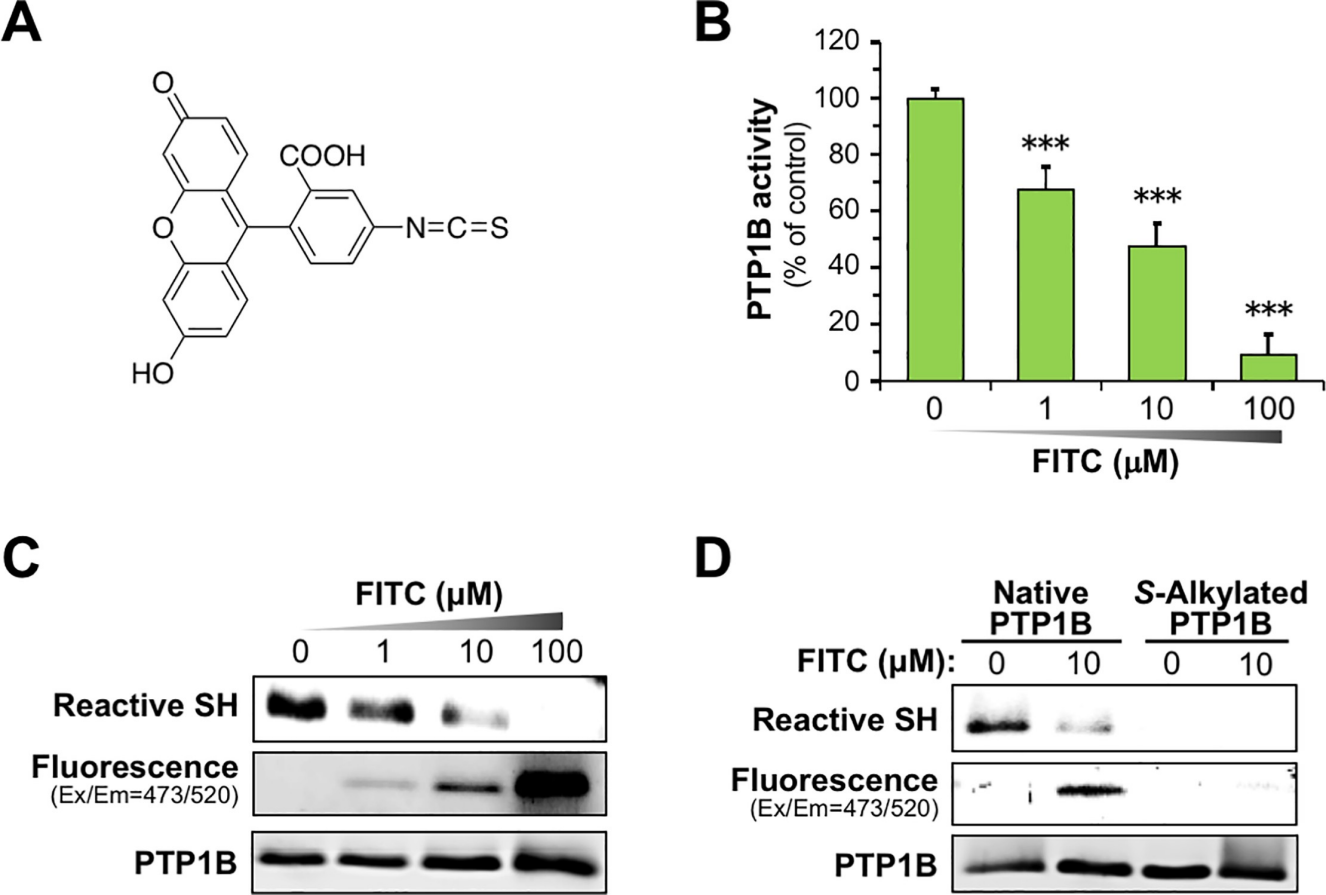
FITC inhibits PTP1B by binding to cysteinyl thiol. (**A**) The structure of FITC. (**B**) Concentration-dependent inhibition of PTP1B activity by FITC. Recombinant PTP1B was incubated with the indicated concentration of FITC in HEPES buffer (pH 7.5) at 37°C for 30 min, and then PTP1B activity was measured. The results are shown as means ± S.E.M. (*n* = 3). ***p < 0.001 *vs* vehicle-treated control (ANOVA, Dunnett’s multiple comparison test). (**C**) Concentration-dependent modification of PTP1B by FITC. Recombinant PTP1B was incubated with the indicated concentration of FITC in HEPES buffer (pH 7.5) at 37°C for 30 min. The reactive thiol group in PTP1B was determined by biotin-labeling assay. The FITC-modified PTP1B was detected by fluorescent imaging. The total PTP1B level was determined by immunoblotting. Data are representative of two independent experiments. (**D**) Cysteine-targeted modification of PTP1B by FITC. Intact and *S*-carbamidomethylated recombinant PTP1B were incubated with or without 10 μM FITC in HEPES buffer (pH 7.5) at 37°C for 30 min. Then, the reactive thiol group, FITC-modified PTP1B, and total PTP1B were determined. Data are representative of two independent experiments.

As shown in **Fig 1B**, the incubation of PEITC with recombinant PTP1B at 37°C for 30 min caused a concentration-dependent reduction in the enzymatic activity with a half-maximal inhibitory concentration (IC_50_) value of 26.2 ϋM. Recent studies have revealed that PTP1B activity is subject to regulation by post-translational modification such as oxidation and alkylation of a reactive deprotonated cysteine residue at the catalytic center [37,38,42]. Accordingly, as illustrated in **Fig 1C**, we speculated that this inactivation could be due to the reaction of ITC with the cysteinyl thiols to form an unstable thiocarbamoyl adduct. To examine whether or not the active site of PTP1B is modified by PEITC, we determined the reactive cysteinyl thiol in PTP1B using the cysteine-reactive probe iodoacetyl-LC-Biotin [38]. After labeling the reactive thiol with iodoacetyl-LC-Biotin, the incorporation of biotin-tagged probe into PTP1B was detected with HRP-conjugated streptavidin. As expected, the incubation of PEITC with PTP1B for 30 min resulted in a concentration-dependent loss of the reactive cysteinyl thiol (**Fig 1D** and **S1A Fig**). Moreover, we also observed that BITC, PITC, and AITC inactivate PTP1B in a concentration-dependent manner, and cause a significant decrease in the reactive cysteinyl thiol (**S1B–S1D Fig**). To further verify whether or not ITC directly inactivates PTP1B by the covalent modification, we fluorometrically assessed the modification of PTP1B by a fluorescent FITC (**Fig 2A**). We confirmed that FITC inhibits PTP1B activity in a concentration-dependent manner (**Fig 2B**). As shown in **Fig 2C** and **S2A Fig**, the incubation of PTP1B with FITC elicited a dose-dependent increase in the fluorescent intensity of PTP1B with a concomitant decrease in the reactive cysteinyl thiol. Furthermore, to obtain definitive evidence that FITC binds to the cysteinyl thiol of PTP1B, we prepared *S*-carbamidomethylated PTP1B by alkylation of free cysteine residues with iodoacetamide. As shown in **Fig 2D** and **S2B Fig**, compared with intact PTP1B, incubation of *S*-carbamidomethylated PTP1B with FITC drastically reduced the fluorescent intensity of PTP1B. These results suggest that ITCs inactivate PTP1B by binding to the catalytic cysteinyl thiol.

### PEITC inhibits cellular PTP1B and activates leptin signaling in differentiated neuronal SH-SY5Y cells

To investigate the effect of PEITC on leptin intracellular signaling, we used differentiated neuronal SH-SY5Y cells, which represent a suitable cellular model with a*t*RA-induced Ob-Rb expression [39]. As shown in **S3 Fig**, we validated that treatment of SH-SY5Y neuroblastoma cells with 100 nM RA for 5 days induces increased expression of Ob-Rb and augments neurite outgrowth. Moreover, treatment of differentiated SH-SY5Y cells with leptin functionally stimulated the tyrosine phosphorylation of Ob-Rb, JAK2, and STAT3 (**S4A Fig**). In good agreement with this response, POMC mRNA expression, determined by qRT-PCR, was significantly elevated by leptin stimulation, whereas both NPY and AgRP mRNA expression was reduced (**S4B–S4D Fig**).

**Fig 3:**
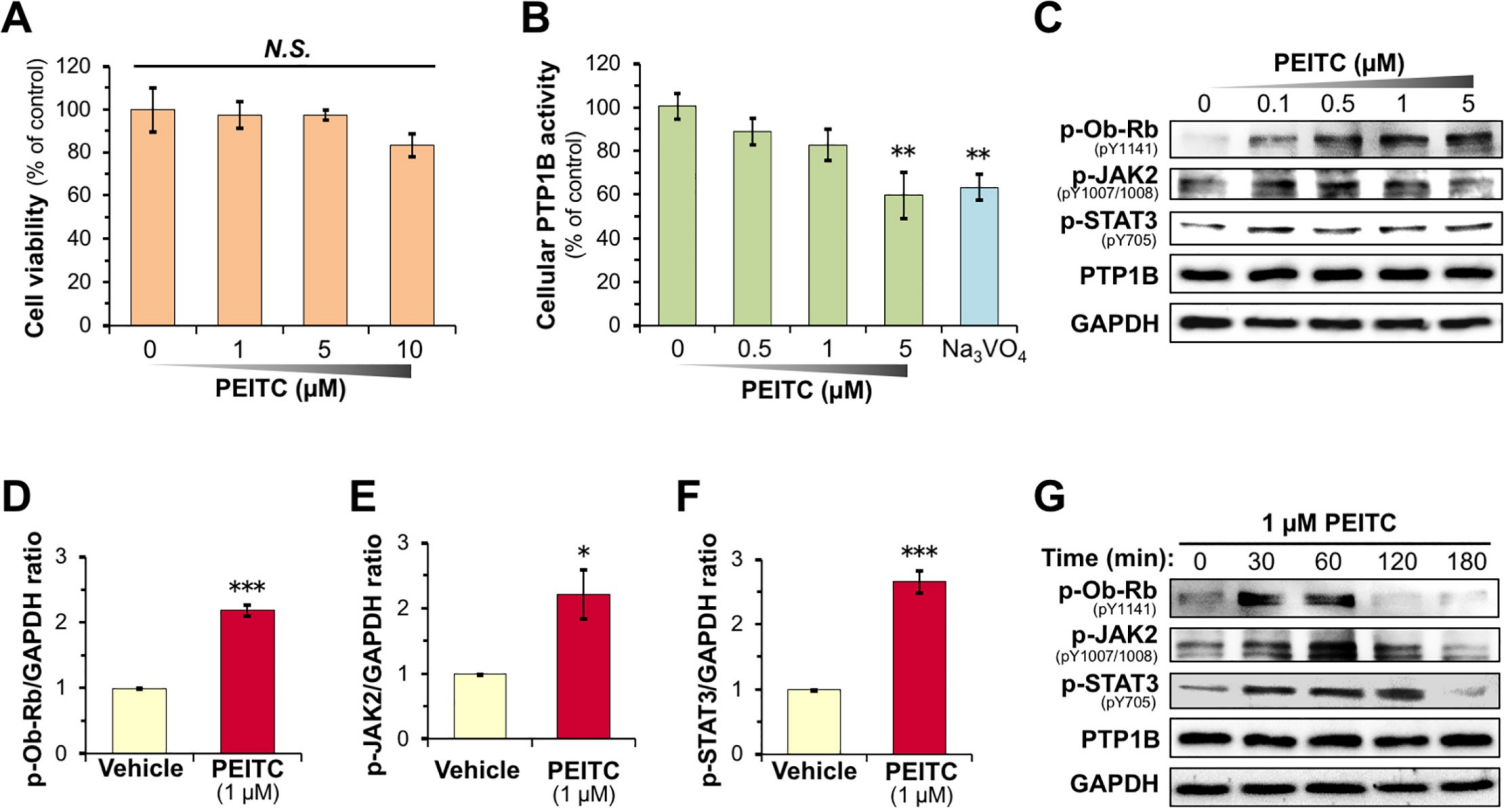
PEITC activates leptin signaling in differentiated SH-SY5Y cells. (**A**) Effects of PEITC treatment on cell viability in the differentiated SH-SY5Y cells. After the SH-SY5Y cells were incubated with the indicated concentration of PEITC in serum-free DMEM for 24 h, the cell viability was measured. The results are shown as means ± S.E.M. (*n* = 4). *N*.*S*. not significant (ANOVA, Dunnett’s multiple comparison test). (**B**) Concentration-dependent inhibition of cellular PTP1B by PEITC treatment in the differentiated SH-SY5Y cells. Cellular PTP1B activity was measured in the SH-SY5Y cells after treatment with the indicated concentrations of PEITC or 200 μM Na_3_VO_4_ in serum-free DMEM for 30 min. The results shown are means ± S.E.M. (*n* = 6). **p < 0.01 *vs* vehicle-treated control (ANOVA, Dunnett’s multiple comparison test). (**C–G**) Activation of leptin signaling in the differentiated SH-SY5Y cells by PEITC treatment. The SH-SY5Y cells were treated with the indicated concentrations of PEITC for 30 min (**C–F**) in serum-free DMEM. Then, the levels of p-Ob-Rb, p-JAK2, p-STAT3, PTP1B, and GAPDH were determined by immunoblotting. (**D–F**) The levels of p-Ob-Rb (**D**), p-JAK2 (**E**), p-STAT3 (**F**) were normalized for GAPDH. Data are the mean ± S.E.M. of three independent immunoblots (**Fig 3C** and **S5A Fig**). *p < 0.05, ***p < 0.001 *vs* vehicle-treated control (Student’s *t*-test). (**G**) Time course of activation of leptin signaling in the differentiated SH-SY5Y cells by PEITC treatment. The SH-SY5Y cells were treated with the indicated concentrations of 1 μM PEITC for 0–180 min in serum-free DMEM. Then, the levels of p-Ob-Rb, p-JAK2, p-STAT3, PTP1B, and GAPDH were determined by immunoblotting. Data are representative of two independent experiments.

After establishing the model SH-SY5Y cell system we assessed cellular responses to PEITC treatment. First, we ascertained that PEITC showed no cytotoxicity against differentiated SH-SY5Y cells at a concentration up to 10 μM over a period of 24 h (**Fig 3A**). To examine whether or not PEITC inactivates cellular PTP1B, we measured PTP1B activity in PEITC-treated differentiated SH-SY5Y cells using a phosphorylated peptide substrate. As shown in **Fig 3B**, the treatment of differentiated SH-SY5Y cells with PEITC for 30 min induced concentration-dependent inhibition of cellular PTP1B activity like the Na_3_VO_4_, a PTP1B inhibitor [43]. Previous studies have proven that PTP1B dephosphorylates a variety of receptor tyrosine kinases (RTKs) including the activated epidermal growth factor receptor (EGFR) and insulin receptor (IR) [44][45]. Intriguingly, the inhibition of PTP1B has been reported to evoke ligand-independent autophosphorylation of EGFR and IR, and activate downstream EGFR and insulin signaling, respectively [36–38,42]. Hence, we investigated whether the inhibition of cellular PTP1B by PEITC in differentiated SH-SY5Y cells provokes a ligand-independent activation of leptin signaling. As shown in **Fig 3C** and **S5A Fig**, we observed that the exposure of differentiated SH-SY5Y cells to PEITC for 30 min resulted in a concentration-dependent phosphorylation of Ob-Rb, JAK2, and STAT3 but had no influence on the protein levels of PTP1B. The densitometric analysis of the bands showed a significant increase in the level of phosphorylated Ob-Rb, JAK2, and STAT3 by treatment of differentiated SH-SY5Y with 1 μM PEITC for 30 min (**Fig 3D–3F**). The leptin-independent activation of Ob-Rb was induced from 30 to 60 min after the exposure with 1 μM PEITC (**Fig 3G and S5B Fig**). On the other hand, the phosphorylation of JAK2 and STAT3 was observed for up to 120 min after the treatment with PEITC. We also confirmed that Na_3_VO_4_ led to a ligand-independent activation of leptin signaling (**S5C Fig**). Thus, our results indicate that PEITC can cause a leptin-independent activation of leptin signaling through PTP1B inhibition. Additionally, we also observed that PEITC potentiates the ligand-dependent activation of leptin signaling in differentiated SH-SY5Y cells (**S5D Fig**).

### PEITC treatment promotes nuclear accumulation of p-STAT3 and regulates mRNA expression of POMC, NPY, and AgRP in differentiated SH-SY5Y cells

**Fig 4:**
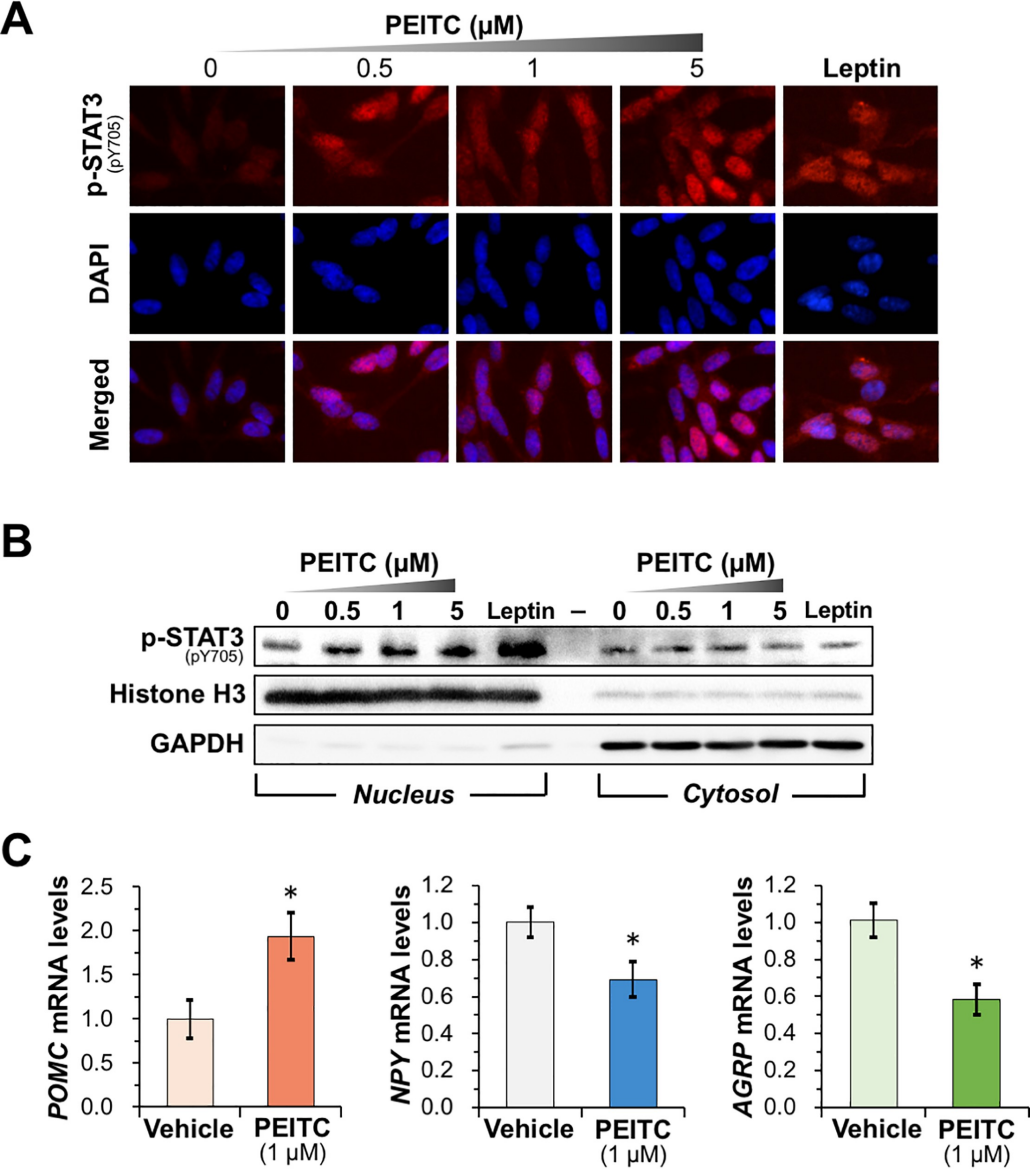
PEITC induces nuclear accumulation of p-STAT3 and regulates its target gene expression in differentiated SH-SY5Y cells. (**A and B**) Nuclear accumulation of p-STAT3 by treatment of the differentiated SH-SY5Y cells with PEITC. The SH-SY5Y cells were incubated with the indicated concentrations of PEITC or 100 nM leptin for 30 min. (**A**) After incubation, p-STAT3 was immunostained by using the anti-p-STAT3 antibody as red, the nuclei were stained with DAPI as blue, and the merged image of p-STAT3 and nuclei as purple. (**B**) After cell fractionation, the nuclear and cytoplasmic levels of p-STAT3 were determined by immunoblotting. The antibodies against histone H3 and GAPDH were used as the loading controls for the nuclear and cytoplasmic protein, respectively. Data are representative of two independent experiments. (**C**) Levels of *POMC*, *NPY*, and *AGRP* mRNA in PEITC-treated differentiated SH-SY5Y cells. The SH-SY5Y cells were treated with 1 μM PEITC for 30 min in serum-free DMEM. The mRNA levels of *POMC*, *NPY*, and *AGRP* were then analyzed by qRT-PCR. The results are shown as means ± S.E.M. (*n* = 3). *p < 0.05 *vs* vehicle-treated control (Student’s *t*-test).

We next examined whether PEITC-inducible activation of leptin signaling promotes nuclear translocation of activated STAT3. As demonstrated by an immunofluorescence microscopic analysis (**Fig 4A**), treatment of differentiated SH-SY5Y cells with PEITC for 30 min resulted in a dose-dependent accumulation of p-STAT3 in the nucleus. This observation was further confirmed by determination of p-STAT3 levels in nuclear and cytosolic fraction by immunoblotting. As shown in **Fig 4B** and **S5E Fig**, our results clearly indicated that the exposure to PEITC increased the nuclear level of p-STAT3 in a concentration-dependent manner but had little effect on the cytosolic level. Furthermore, qRT-PCR analysis revealed that PEITC treatment significantly activates POMC transcription while inhibiting AgRP and NPY transcription (**Fig 4C**). These data suggest that PEITC could reduce food intake and increase energy expenditure by leptin signaling-mediated transcriptional control by inhibiting PTP1B.

### PEITC treatment suppresses food intake during refeeding following fasting in mice

**Fig 5:**
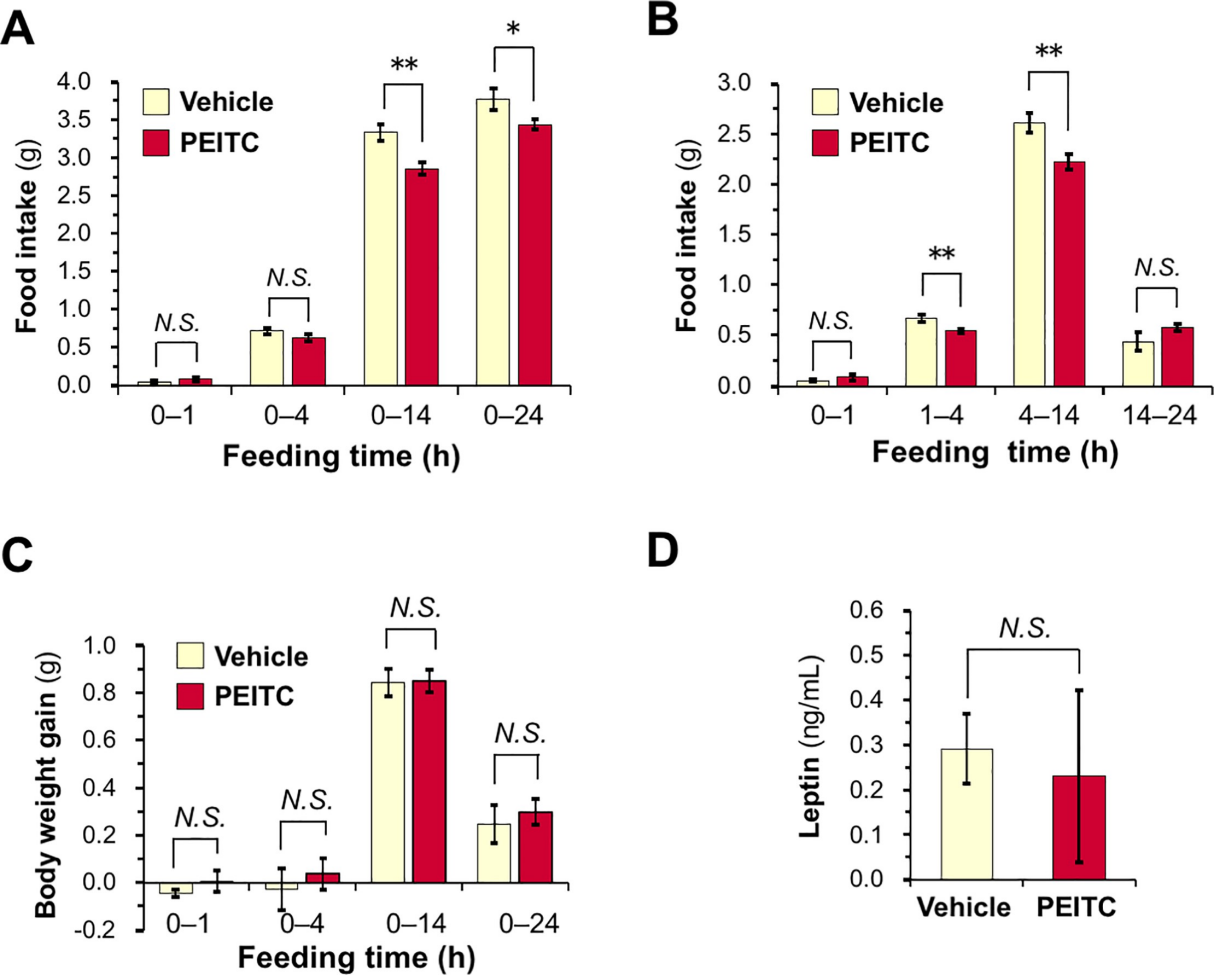
Oral administration of PEITC reduces food intake during refeeding following fasting. (**A–C**) Effects of oral PEITC administration on food intake and body weight. The mice were fasted for 6 h, and orally administered vehicle alone or 25 mg/kg/body weight PEITC. The food was presented after further 1-h fasting. (**A**) Food intake in the next 1, 4, 14, and 24 h were monitored. (**B**) Food intake in the period of 0–1, 1–4, 4–14, and 14–24 h. (**C**) Body weight change following refeeding were monitored. (**D**) The mice were fasted for 6 h, and orally administered vehicle alone or PEITC (25 mg/kg/body weight). After further 2-h fasting, the blood sample was obtained from tail veins, and plasma leptin concentrations were measured. (**A–D**) The results are shown as means ± S.E.M. (*n* = 6 or 7). *N*.*S*., not significant. *p < 0.05, **p < 0.01 *vs* vehicle-treated control (Student’s *t*-test).

**Fig 6:**
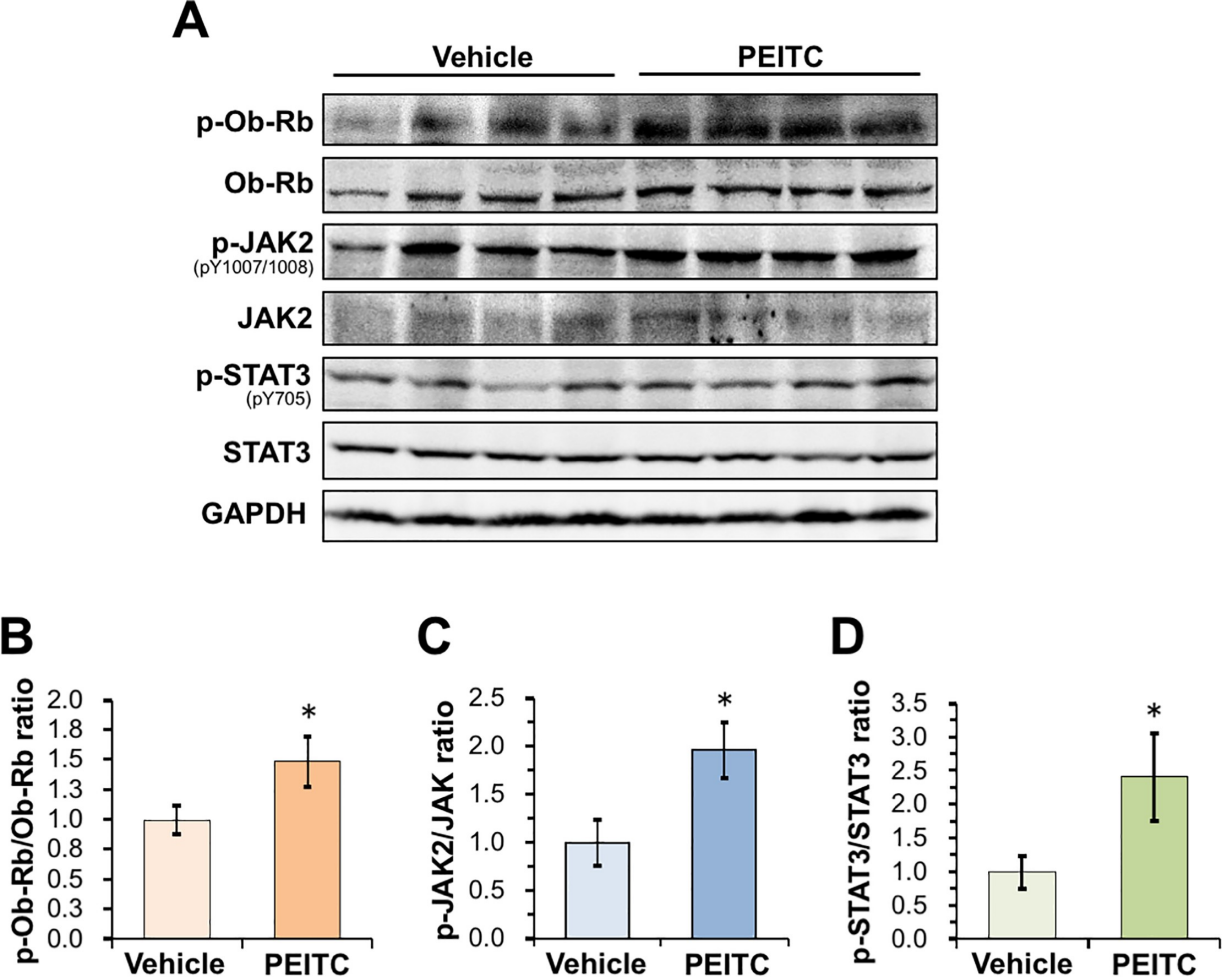
Oral administration of PEITC stimulates leptin signaling in the hypothalamus. (**A–C**) Effects of oral PEITC administration on leptin signaling in the hypothalamus. The mice were fasted for 6 h, and orally administered vehicle alone or 25 mg/kg/body weight PEITC, followed by further 3-h fasting. Then, the hypothalami were quickly dissected out and extracted. (**A**) The phosphorylation of Ob-Rb, JAK2, and STAT3 in the hypothalamic extracts was detected by immunoblotting. (**B–D**) The levels of p-Ob-Rb/Ob-Rb (**B**), p-JAK2/JAK2 (**C**), and p-STAT3/STAT3 (**D**) were calculated, respectively, and expressed as fold change against vehicle-treated control. The results shown are means ± S.E.M. (*n* = 4). *p < 0.05 *vs* vehicle-treated control (Student’s *t*-test).

To demonstrate *in vivo* potency, we assessed the acute anorexigenic effect of PEITC on food intake during refeeding in mice. After 6-h fasting, the mice were orally administered PEITC (25 mg/kg/body weight) or the vehicle, and further deprived of food for 1 h. Then, the mice were refed with a powdered diet (standard rodent chow). As shown in **Fig 5A**, the oral administration of PEITC significantly reduced 14- and 24-h food intake after refeeding, compared with vehicle control. The anorexigenic effect of PEITC was observed from 1 h to 14 h after refeeding (**Fig 5B**). On the other hand, PEITC treatment did not significantly decrease body weight gain during 24-h refeeding, compared with vehicle treatment (**Fig 5C**). We also observed that there was no significant difference in plasma leptin levels 2 h after the administration between the vehicle and PEITC groups (**Fig 5D**). Our data demonstrated a rapid effect of feeding inhibition (as early as 2 h post-administration) by oral treatment of PEITC. Furthermore, we evaluated the phosphorylation levels of Ob-Rb, JAK2, and STAT3 in the hypothalamus 3 h after the oral administration by immunoblotting. As shown in **Fig 6A–6D**, PEITC administration caused significant increases in the phosphorylation levels of Ob-Rb, JAK2, and STAT3 in the hypothalamus compared with the vehicle control. These data suggest that PEITC may decrease food intake by stimulating the leptin signaling *via* PTP1B inhibition.

## Discussion

Most obese subjects develop leptin resistance and hyperleptinemia, restricting the therapeutic efficacy of leptin treatment. Targeting molecules required for the development of leptin resistance is a potentially effective therapeutic approach for preventing the obesity epidemic. Accumulating evidence from animal models, clinical and cellular studies suggest that PTP1B could be involved in the pathways leading to leptin resistance as a major negative regulator of leptin signaling. A recent study revealed that hypothalamic PTP1B levels in rats increase coincident with the initial development of leptin resistance, and that intracerebroventricular administration of a pharmacological competitive PTP1B inhibitor restores leptin sensitivity, resulting in a dose-dependent inhibition of food intake [15]. Studies in cell culture also revealed an inverse relationship between PTP1B expression and leptin sensitivity [9–11]. Importantly, PTP1B-deficient mice display the resistance to diet-induced obesity, augmented energy expenditure, hypersensitivity to leptin, and enhanced phosphorylation levels of JAK2 and STAT3 in the hypothalamus [10,46,47]. These mice also exhibited an augmented sensitivity to insulin with increased tyrosyl phosphorylation of the IR in the liver and muscle. It is notable that PTP1B-null mice have no apparent disease phenotype and have a normal fetal survival rate [46,47]. Therefore, it is anticipated that an orally absorbable PTP1B inhibitor would demonstrate anti-obesity effects by enhancing leptin sensitivity in obese subjects. Furthermore, the medication of other diseases such as type 2 diabetes, infectious, autoimmune, or neurological disorders, and cancer is also expected to benefit from PTP1B inhibitor research [48–50]. In the study presented here, we indeed validated that PEITC, a constituent of many cruciferous vegetables, potently inhibits cellular PTP1B activity, resulting in the stimulation of leptin signaling in differentiated neuronal SH-SY5Y cells (**Figs 3 and 4**). Moreover, we also demonstrated that the oral administration of PEITC to mice induced enhanced phosphorylation of Ob-Rb, JAK2, and STAT3 in the hypothalamus, and significantly reduced food intake (**Figs 5 and 6**). Our current findings provide, for the first time, insight into the preventive implications of an orally available PTP1B inhibitor, PEITC, for obesity.

**Fig 7:**
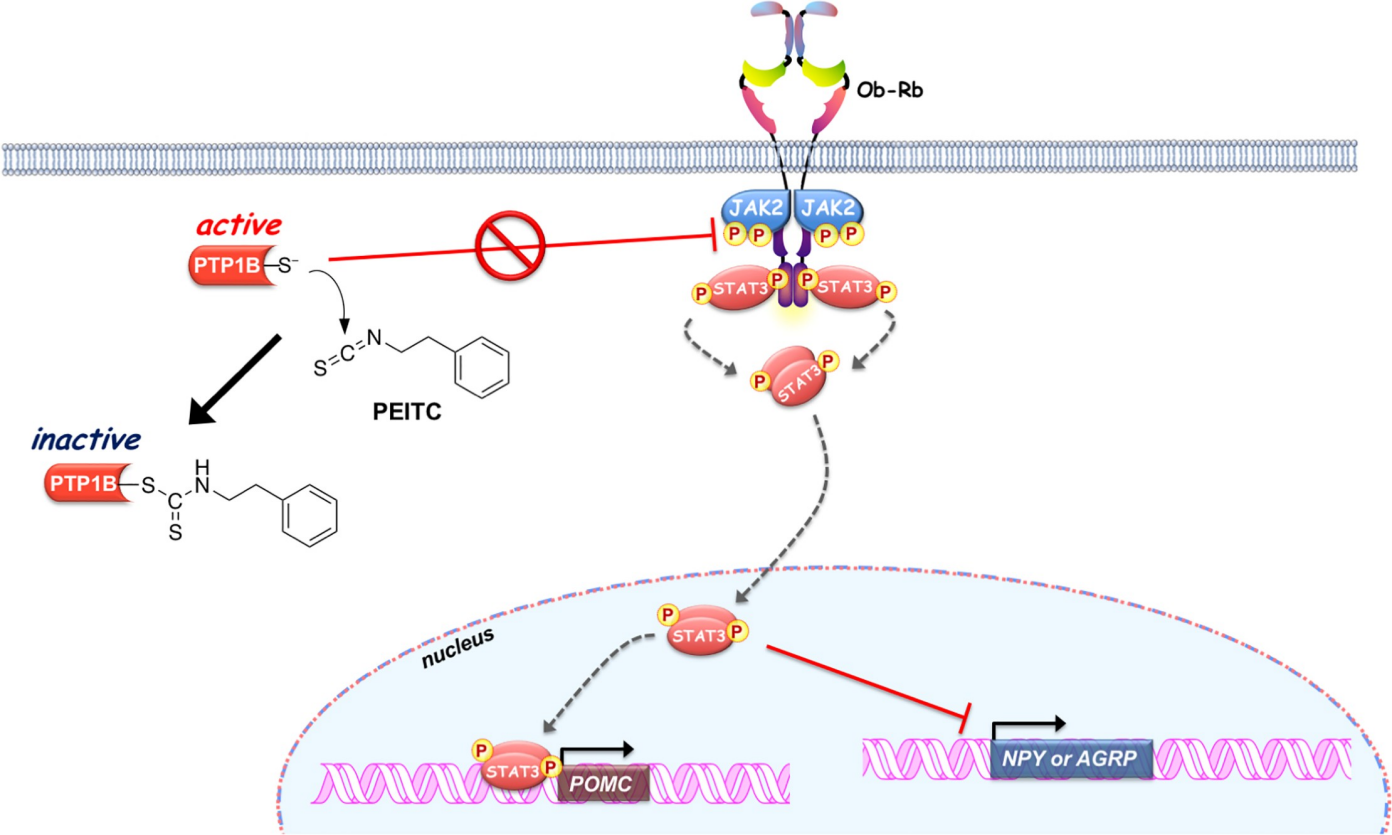
Proposed mechanism by which PEITC activates the leptin signaling pathway *via* inhibiting the cellular PTP1B to induce feeding suppression.

In the present study, we also characterized the molecular mechanism underlying PEITC-mediated inhibition of PTP1B as summarized in **Fig 7**. PTP1B consists of 435 amino acids with a molecular weight of approximately 50 kDa, and is localized on the cytoplasmic face of the endoplasmic reticulum by means of a hydrophobic C-terminal sequence (35 residues) [51]. Due to the unique chemical environment of the phosphatase active site, the catalytic Cys-215 that is located at the base of the active site cleft, has an unusually low acid dissociation constant (p*K*_a_) (~5.4), making it a thiolate anion (Cys–S^−^) at a physiological pH [29]. The low p*K*_a_ enhances the catalytic function of the Cys-215 as a nucleophile but renders it susceptible to oxidation or electrophilic alkylation [48,49,52]. Thus, post-translational modifications of PTP1B are key modulators of its enzymatic activity, and are considered to provoke various physiological responses *via* facilitated tyrosine phosphorylation of signaling proteins. With this reactivity in mind, we demonstrated that ITCs caused a dose-dependent modification of the reactive cysteinyl thiol in PTP1B (**Figs 1 and 2, and S1 Fig**). These results strongly suggest that ITCs inactivate PTP1B by covalent binding to the catalytic cysteinyl thiol. To determine the modification site by PEITC, the native and PEITC-treated PTP1B were digested with V8 and trypsin and then analyzed by ultra-performance liquid chromatography time-of-flight mass spectrometry (UPLC-TOF-MS) [27]. However, we could not observe any modified peptides, probably due to instability of the thiocarbamoyl adduct [53]. Recently, we found that safranal, a doubly unsaturated aldehyde isolated from saffron, effectively inhibited PTP1B by covalent addition to the reactive cysteine through a Michael reaction [38]. Safranal was also demonstrated to elicit an insulin-independent activation of insulin signaling and enhance glucose uptake in C2C12 myotubes. Furthermore, we revealed that oral administration of safranal improved impaired glucoses tolerance in type 2 diabetic KK-*A*^*y*^ mice. In the meantime, several quinone compounds including 1,2-naphthoquinone and pyrroloquinoline quinone have been reported to inhibit PTP1B activity through the oxidation and/or covalent modification of the active site cysteinyl thiol [54]. These quinones cause the ligand-independent activation of IR and/or EGFR and its downstream signaling in epithelial cells, keratinocytes, or myotubes. Hence, inactivation of PTP1B would shift the tyrosine phosphorylation/dephosphorylation equilibrium towards phosphorylation since much lower tyrosine kinase activity will now suffice to evoke receptor tyrosine kinase phosphorylation and activation in the absence of the ligand [29]. In this study, we found that inhibition of cellular PTP1B by PEITC or Na_3_VO_4_ gives rise to the leptin-independent phosphorylation of Ob-Rb, JAK2, and STAT3 in differentiated SH-SY5Y cells (**Figs 3 and 4, and S5 Fig**). JAK2 associates with the cytoplasmic domains of Ob-Rb, and leptin-induced receptor rearrangement facilitates JAK2 trans-phosphorylation of activation-loop Tyr-1007 and Tyr-1008 in JH1 (the tyrosine kinase domain), leading to receptor phosphorylation and recruitment and phosphorylation of STAT3 [55]. Notably, mouse embryo fibroblasts deficient in PTP1B have been shown to display hyperphosphorylation of JAK2 on Tyr-1007/Tyr-1008 and STAT3 on Tyr-705 [56]. This observation illustrates that the tyrosine phosphorylation of JAK2 is tightly regulated by PTP1B maintaining a minimal level in the absence of ligand. Therefore, the ligand-independent activation of leptin signaling by PEITC may be attributed to the basal JAK2 kinase activity emerged by inactivation of PTP1B, shown in **Fig 7**.

In humans, the amount of glucosinolates consumed is estimated to be about 300 mg/day from various cruciferous vegetables [57], and approximately 2 to 6 mg of PEITC is released per ounce (28.3 g) of watercress consumed [58]. PEITC is highly bioavailable after oral administration because of low clearance as well as high protein binding [59]. A single oral dose of 10–100 μmol/kg PEITC in rats resulted in bioavailability ranging between 90 and 114% [59]. Furthermore, the peak plasma concentration of PEITC in human subjects was about 928.5 ± 250 nM, after the consumption of 100 g watercress [60]. The time to reach the peak plasma concentration was 2.6 h ± 1.1 h with a t1/2 4.9 ± 1.1 h. The acute oral LD50 of PEITC for female rats has been determined to be 1.47 g/kg, which shows it has extremely low toxicity [61]. More importantly, an organ distribution study in rats has clarified a fair availability of PEITC in the brain, suggesting better chances of PEITC to cross blood-brain barrier [35]. Our results presented here demonstrate that the exposure of differentiated SH-SY5Y cells to a physiologically relevant concentration of PEITC significantly activates leptin signaling (**Figs 3 and 4**). Accordingly, these observations indicate that oral administration of PEITC can result in effective concentrations in the brain. Indeed, we revealed that the oral administration of PEITC (25 mg/kg/body weight) stimulated hypothalamic leptin signaling, and significantly reduced food intake (**Figs 5 and 6**). Hence, PEITC administration including the high ingestion of cruciferous vegetables can be expected to be beneficial for preventing and improving obesity without adverse effects. However, long-term *in vivo* studies are required to establish the anti-obesity efficacy, efficient dosage, and safety profile of PEITC. In the future, we must adequately assess the long-term potency of PEITC ingestion for suppression of food intake and body weight gain, and improvement of leptin responsiveness in obese mice fed a high-fat diet or in obese human subjects.

## Supporting information

S1 Fig Food-derived ITCs inhibit PTP1B with concomitant decrease in cysteinyl thiol. (**A**) Concentration-dependent modification of cysteinyl thiol in PTP1B by PEITC. Recombinant PTP1B was incubated with the indicated concentration of PEITC in HEPES buffer (pH 7.5) at 37°C for 30 min. Then, the reactive thiol group and total PTP1B were determined by biotin-labeling assay and immunoblotting, respectively. (**B**) The structures of food-derived ITCs. (**C**) The concentration-dependent inhibition of PTP1B activity by ITCs. Recombinant PTP1B was incubated with the indicated concentration of ITC in HEPES buffer (pH 7.5) at 37°C for 30 min, and then PTP1B activity was measured. The results are shown as means ± S.E.M. (*n* = 3). (**D**) Concentration-dependent modification of cysteinyl thiol in PTP1B by ITCs. Recombinant PTP1B was incubated with the indicated concentration of ITC in HEPES buffer (pH 7.5) at 37°C for 30 min. Then, the reactive thiol group and total PTP1B were determined.
(TIFF)

S2 Fig FITC binds to cysteinyl thiol in PTP1B. (**A**) Concentration-dependent modification of PTP1B by FITC. Recombinant PTP1B was incubated with the indicated concentration of FITC in HEPES buffer (pH 7.5) at 37°C for 30 min. (**B**) Cysteine-targeted modification of PTP1B by FITC. Intact and *S*-carbamidomethylated recombinant PTP1B were incubated with or without 10 μM FITC in HEPES buffer (pH 7.5) at 37°C for 30 min. (**A and B**) The reactive thiol group in PTP1B was determined by biotin-labeling assay. The FITC-modified PTP1B was detected by fluorescent imaging. The total PTP1B level was determined by immunoblotting.
(TIFF)

S3 Fig Expression of Ob-Rb in SH-SY5Y neuroblastoma cells is increased by a*t*RA-induced differentiation. (**A**) The expression level of Ob-Rb in a*t*RA-exposed SH-SY5Y cells. The cells were differentiated by treatment with 0–100 nM a*t*RA between 1 and 6 days after plating on a collagen-coated culture plate. Then, the levels of Ob-Rb, JAK2, STAT3, and GAPDH were determined by immunoblotting. (**B**) The mRNA expression levels of Ob-Rb in the undifferentiated and differentiated SH-SY5Y cells. The RT-PCR was performed on undifferentiated cells and differentiated cells following the exposure to 100 nM a*t*RA for 5 days using PrimeScript One Step RT-PCR Kit Ver.2 (Takara Bio). Primers for the human *OB-RB* (Forward 5'-CCTCTTCCATCTTATTGCTTGGA, reverse 5’-CTCAAACGTTTCTGGCTTCTGAA) was purchased from Thermo Fisher Scientific. Primers for human *ribosomal protein S18* (*RPS18*) (Primer Set ID: HA067807) were purchased from Takara Bio. (**C**) Phase-contrast images of the undifferentiated cells (left panel) and differentiated cells following the exposure to 100 nM a*t*RA for 5 days (right panel).
(TIFF)

S4 Fig Leptin stimulates phosphorylation of Ob-Rb, JAK2, and STAT3, and regulates mRNA expression of *POMC*, *NPY*, and *AGRP* in differentiated SH-SY5Y cells. (**A and B**) The SH-SY5Y cells were treated with 100 nM leptin for 30 min in serum-free DMEM. (**A**) Activation of leptin signaling in leptin-stimulated differentiated SH-SY5Y cells. After leptin treatment, the levels of p-Ob-Rb, p-JAK2, p-STAT3, and GAPDH were determined by immunoblotting. (**B**) Levels of *POMC*, *NPY*, and *AGRP* mRNA in leptin-stimulated differentiated SH-SY5Y cells. After leptin treatment, mRNA levels of *POMC*, *NPY*, and *AGRP* were analyzed by qRT-PCR. The results are shown as means ± S.E.M. (*n* = 3). *p < 0.05, **p < 0.01 *vs* vehicle-treated control (Student’s *t*-test).
(TIFF)

S5 Fig PEITC stimulates phosphorylation of Ob-Rb, JAK2, and STAT3 in differentiated SH-SY5Y cells. (**A and B**) Activation of leptin signaling in the differentiated SH-SY5Y cells by PEITC treatment. The SH-SY5Y cells were treated with the indicated concentrations of PEITC for 30 min (**A**) or 0–120 min (**B**) in serum-free DMEM. (**C**) Activation of leptin signaling in the differentiated SH-SY5Y cells by Na_3_VO_4_ treatment. The SH-SY5Y cells were treated with 100 nM leptin for 30 min or with 200 μM Na_3_VO_4_ for 60 min in serum-free DMEM. (**D**) Effect of PEITC on ligand-dependent activation of leptin signaling in the differentiated SH-SY5Y. The SH-SY5Y cells were treated with the indicated concentrations of PEITC in the presence or absence of 50 nM leptin for 30 min. (**E**) Nuclear accumulation of p-STAT3 by treatment of the differentiated SH-SY5Y cells with PEITC. The SH-SY5Y cells were incubated with the indicated concentrations of PEITC or 100 nM leptin for 30 min. Then, nuclear and cytoplasmic fractions were prepared. (**A–E**) The levels of p-Ob-Rb, p-JAK2, p-STAT3, PTP1B, histon H3, and GAPDH were determined by immunoblotting.
(TIFF)

## Abbreviations

AITC: allyl isothiocyanate
AgRP: agouti related peptide
BITC: benzyl isothiocyanate
DAPI: 4´,6-diamidino-2-phenylindole
EGFR: epidermal growth factor receptor
FITC: fluorescein isothiocyanate
GAPDH: glyceraldehyde-3-phosphate dehydrogenase
HEPES: 4-(2-hydroxyethyl)-1-piperazineethanesulfonic acid
IR: insulin receptor
ITCs: isothiocyanates
JAK2: Janus kinase 2
NPY: neuropeptide Y
PEITC: phenethyl isothiocyanate
PITC: phenyl isothiocyanate
POMC: proopiomelanocortin
PTP1B: protein tyrosine phosphatase 1B
RA: all-*trans*-retinoic acid
STAT3: signal transducer and activator of transcription 3
pNPP: *p*-nitrophenol phosphate
