# Supplementary figures and images for: Phenethyl isothiocyanate activates leptin signaling and decreases food intake
Source: PLoS One. 2018 Nov 1;13(11):e0206748. doi: 10.1371/journal.pone.0206748 (PMC6211728; doi:10.1371/journal.pone.0206748)

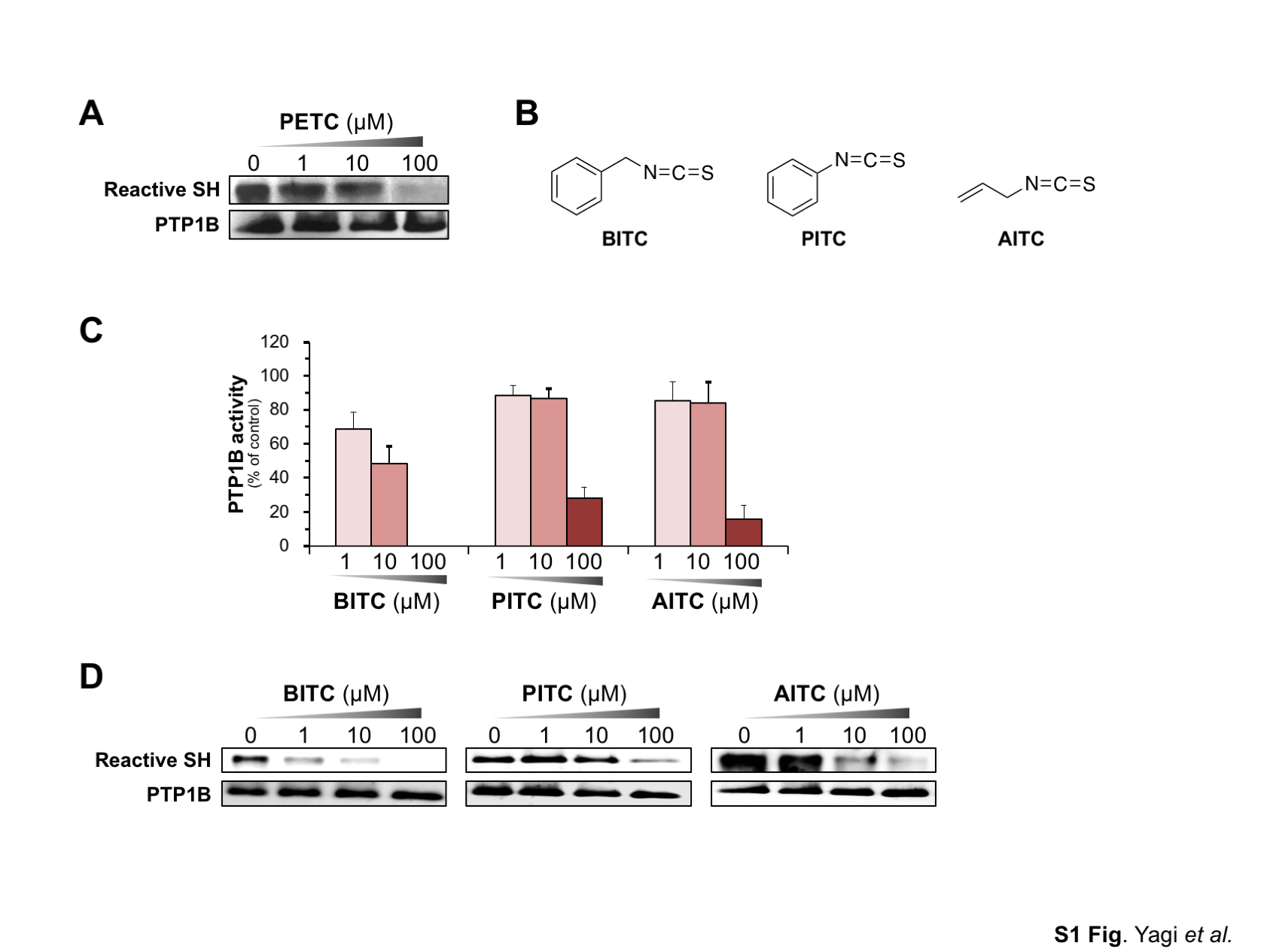

Supplement: S1 Fig — (A) Concentration-dependent modification of cysteinyl thiol in PTP1B by PEITC. Recombinant PTP1B was incubated with the indicated concentration of PEITC in HEPES buffer (pH 7.5) at 37°C for 30 min. Then, the reactive thiol group and total PTP1B were determined by biotin-labeling assay and immunoblotting, respectively. (B) The structures of food-derived ITCs. (C) The concentration-dependent inhibition of PTP1B activity by ITCs. Recombinant PTP1B was incubated with the indicated concentration of ITC in HEPES buffer (pH 7.5) at 37°C for 30 min, and then PTP1B activity was measured. The results are shown as means ± S.E.M. (n = 3). (D) Concentration-dependent modification of cysteinyl thiol in PTP1B by ITCs. Recombinant PTP1B was incubated with the indicated concentration of ITC in HEPES buffer (pH 7.5) at 37°C for 30 min. Then, the reactive thiol group and total PTP1B were determined. (TIFF) [file pone.0206748.s001.tiff]

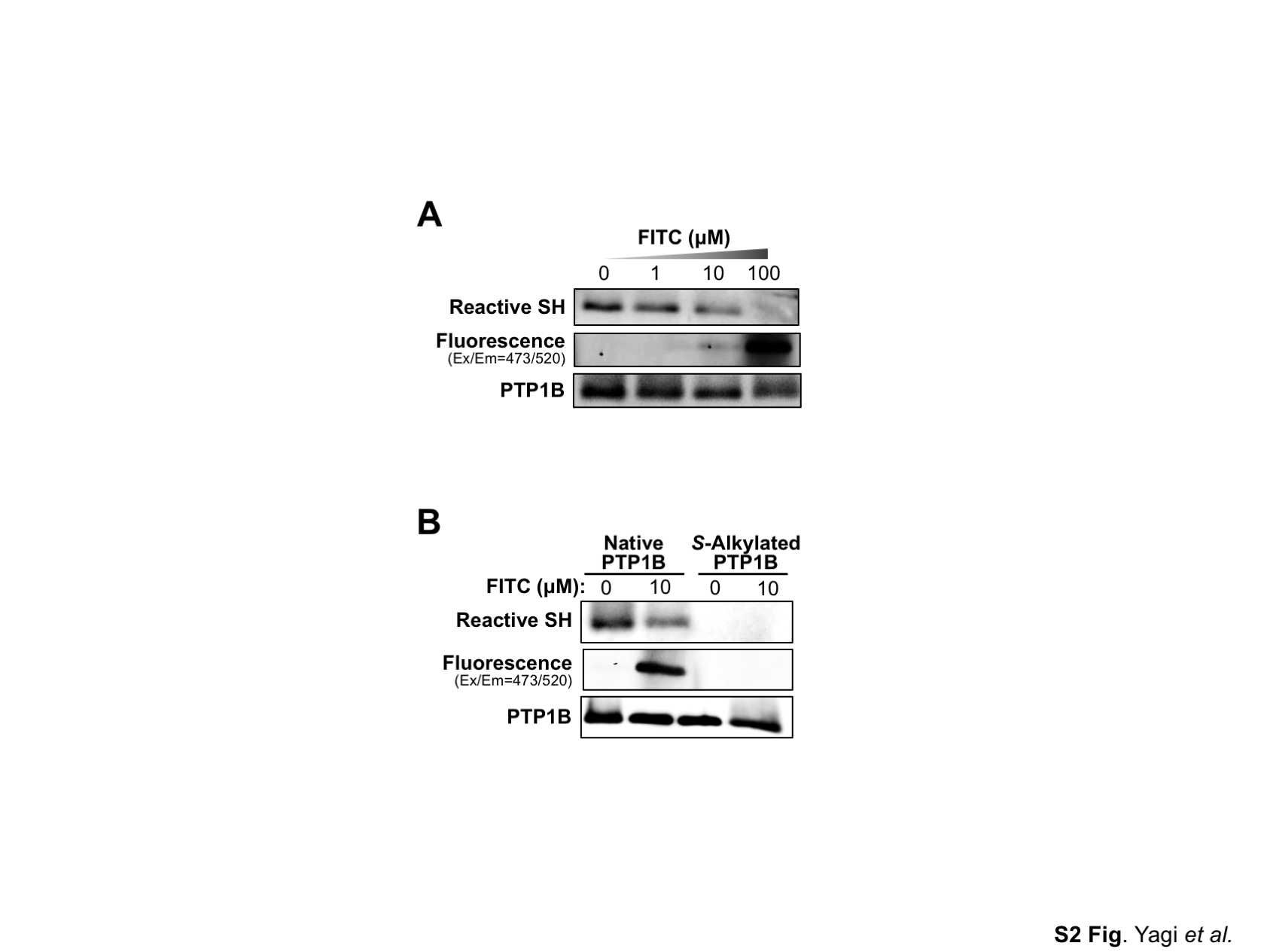

Supplement: S2 Fig — (A) Concentration-dependent modification of PTP1B by FITC. Recombinant PTP1B was incubated with the indicated concentration of FITC in HEPES buffer (pH 7.5) at 37°C for 30 min. (B) Cysteine-targeted modification of PTP1B by FITC. Intact and S-carbamidomethylated recombinant PTP1B were incubated with or without 10 μM FITC in HEPES buffer (pH 7.5) at 37°C for 30 min. (A and B) The reactive thiol group in PTP1B was determined by biotin-labeling assay. The FITC-modified PTP1B was detected by fluorescent imaging. The total PTP1B level was determined by immunoblotting. (TIFF) [file pone.0206748.s002.tiff]

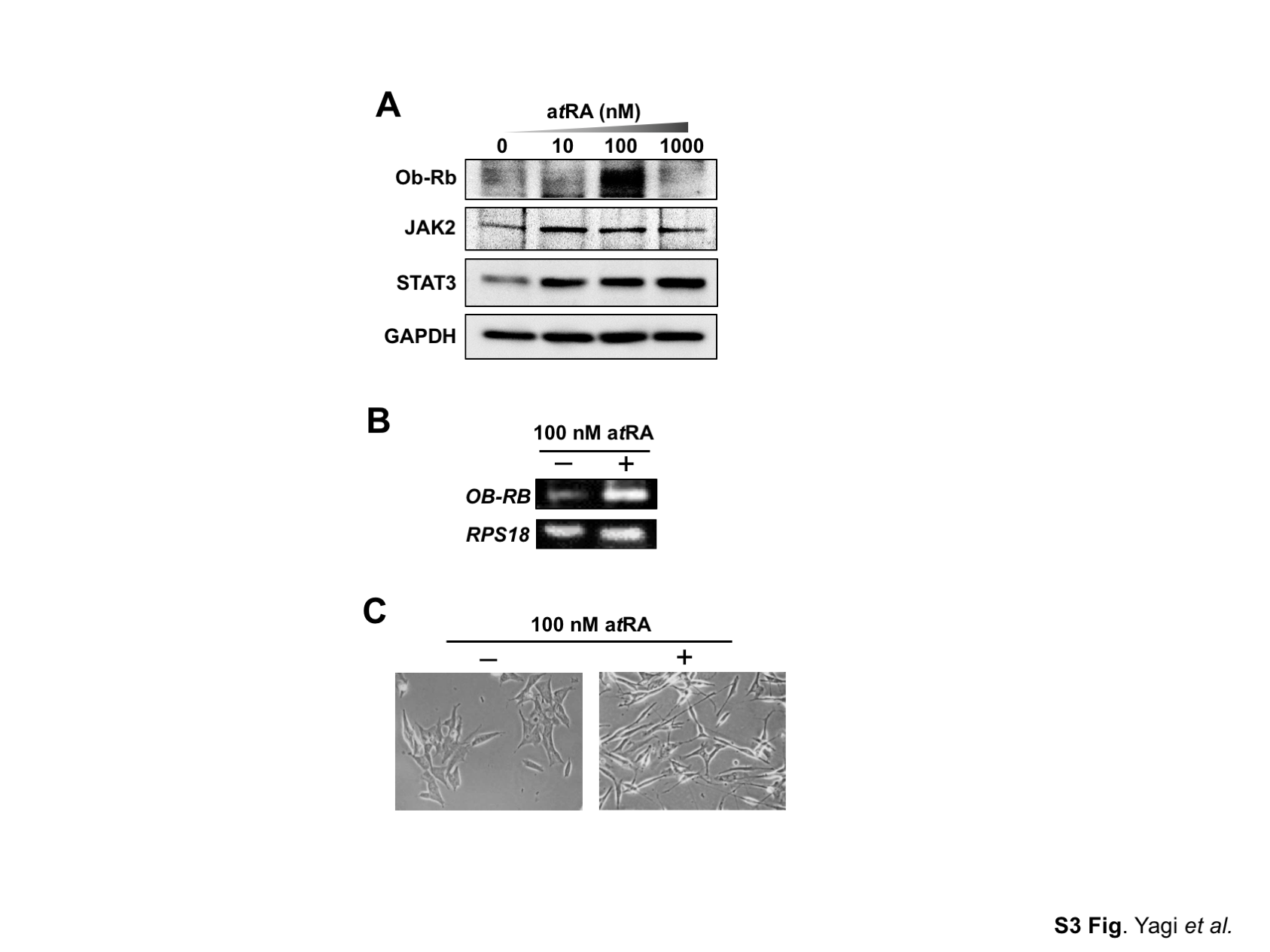

Supplement: S3 Fig — (A) The expression level of Ob-Rb in atRA-exposed SH-SY5Y cells. The cells were differentiated by treatment with 0–100 nM atRA between 1 and 6 days after plating on a collagen-coated culture plate. Then, the levels of Ob-Rb, JAK2, STAT3, and GAPDH were determined by immunoblotting. (B) The mRNA expression levels of Ob-Rb in the undifferentiated and differentiated SH-SY5Y cells. The RT-PCR was performed on undifferentiated cells and differentiated cells following the exposure to 100 nM atRA for 5 days using PrimeScript One Step RT-PCR Kit Ver.2 (Takara Bio). Primers for the human OB-RB (Forward 5'-CCTCTTCCATCTTATTGCTTGGA, reverse 5’-CTCAAACGTTTCTGGCTTCTGAA) was purchased from Thermo Fisher Scientific. Primers for human ribosomal protein S18 (RPS18) (Primer Set ID: HA067807) were purchased from Takara Bio. (C) Phase-contrast images of the undifferentiated cells (left panel) and differentiated cells following the exposure to 100 nM atRA for 5 days (right panel). (TIFF) [file pone.0206748.s003.tiff]

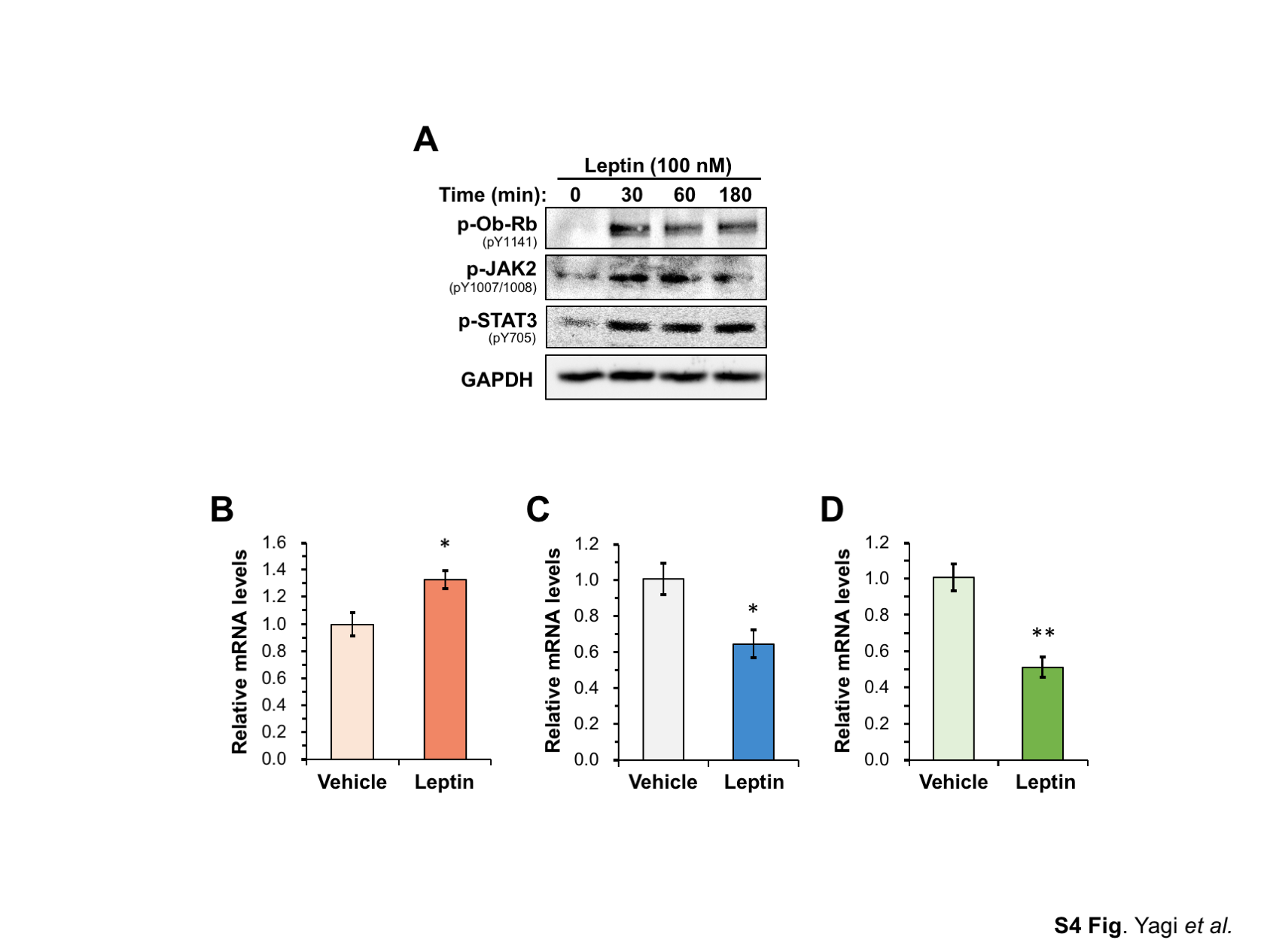

Supplement: S4 Fig — (A and B) The SH-SY5Y cells were treated with 100 nM leptin for 30 min in serum-free DMEM. (A) Activation of leptin signaling in leptin-stimulated differentiated SH-SY5Y cells. After leptin treatment, the levels of p-Ob-Rb, p-JAK2, p-STAT3, and GAPDH were determined by immunoblotting. (B) Levels of POMC, NPY, and AGRP mRNA in leptin-stimulated differentiated SH-SY5Y cells. After leptin treatment, mRNA levels of POMC, NPY, and AGRP were analyzed by qRT-PCR. The results are shown as means ± S.E.M. (n = 3). *p < 0.05, **p < 0.01 vs vehicle-treated control (Student’s t-test). (TIFF) [file pone.0206748.s004.tiff]

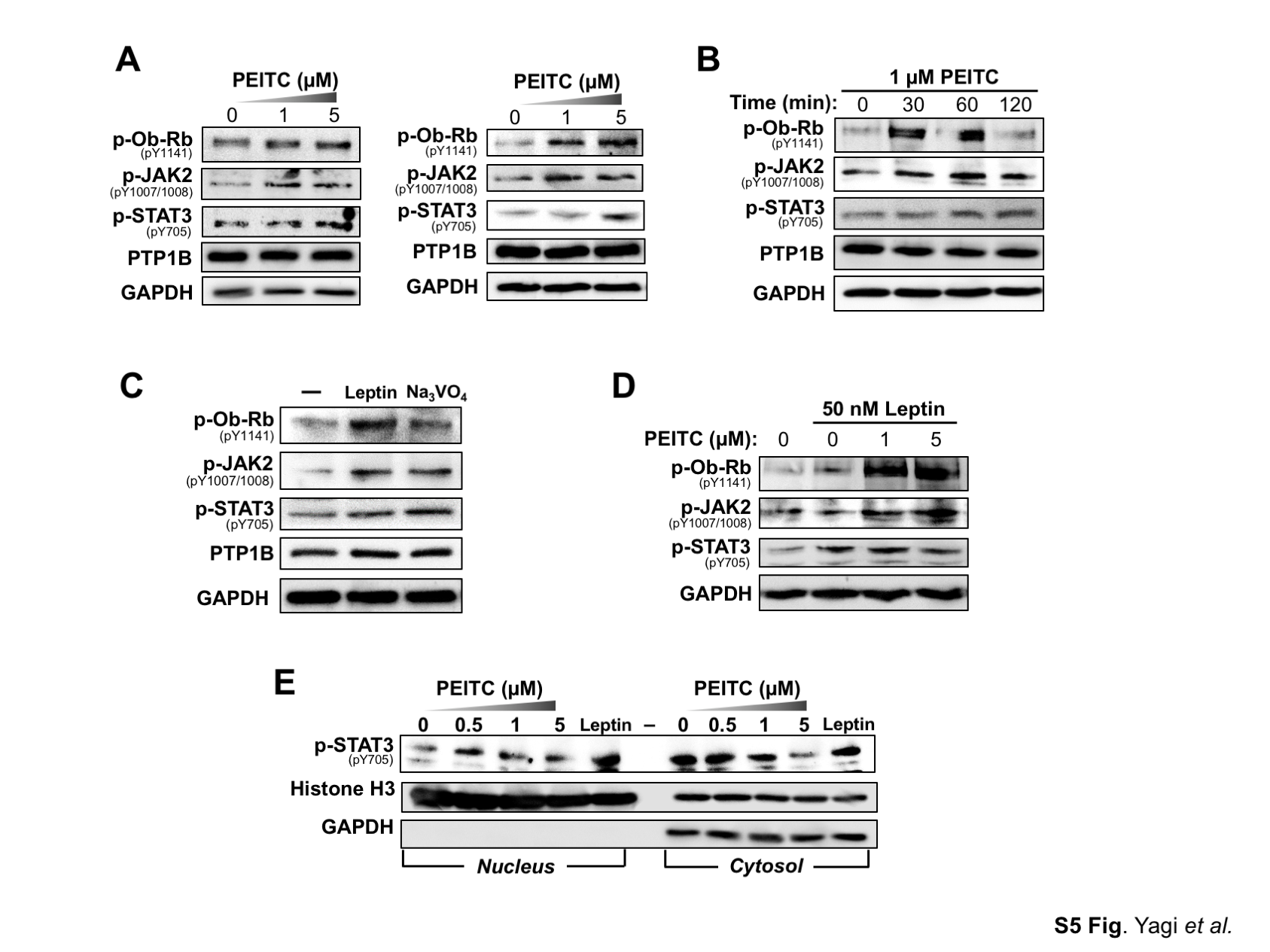

Supplement: S5 Fig — (A and B) Activation of leptin signaling in the differentiated SH-SY5Y cells by PEITC treatment. The SH-SY5Y cells were treated with the indicated concentrations of PEITC for 30 min (A) or 0–120 min (B) in serum-free DMEM. (C) Activation of leptin signaling in the differentiated SH-SY5Y cells by Na3VO4 treatment. The SH-SY5Y cells were treated with 100 nM leptin for 30 min or with 200 μM Na3VO4 for 60 min in serum-free DMEM. (D) Effect of PEITC on ligand-dependent activation of leptin signaling in the differentiated SH-SY5Y. The SH-SY5Y cells were treated with the indicated concentrations of PEITC in the presence or absence of 50 nM leptin for 30 min. (E) Nuclear accumulation of p-STAT3 by treatment of the differentiated SH-SY5Y cells with PEITC. The SH-SY5Y cells were incubated with the indicated concentrations of PEITC or 100 nM leptin for 30 min. Then, nuclear and cytoplasmic fractions were prepared. (A–E) The levels of p-Ob-Rb, p-JAK2, p-STAT3, PTP1B, histon H3, and GAPDH were determined by immunoblotting. (TIFF) [file pone.0206748.s005.tiff]
